# Advancing the assessment of pacifier effects with a novel computational method

**DOI:** 10.1186/s12903-023-03848-5

**Published:** 2024-01-16

**Authors:** R. Pereira, J. Romero, A. Norton, J. M. Nóbrega

**Affiliations:** 1 IPC – Institute for Polymers and Composites, University of Minho, Azurém Campus, Guimarães, 4804-058 Portugal; 2 FMDUP – Faculdade de Medicina Dentária da Universidade do Porto, Porto, 4200-393 Portugal

**Keywords:** Mouth, Deciduous tooth, Sucking behavior, Malocclusion, Computer simulation, Computer-aided engineering

## Abstract

**Background:**

Numerous studies have demonstrated a high likelihood of malocclusions resulting from non-nutritive sucking. Consequently, quantifying the impact of pacifiers can potentially aid in preventing the development or exacerbation of malocclusions and guide the design of improved performance pacifiers.

**Methods:**

This work proposes and assesses a computational methodology that can effectively gather crucial information and provide more precise data regarding the consequences of non-nutritive pacifier sucking. The computational framework utilized is based on solids4Foam [1, 2], a collection of numerical solvers developed within the OpenFOAM® computational library [3]. The computational model focuses on the palate of a six-month-old baby and incorporates various components such as palate tissues, pacifier and tongue, and considers the negative intraoral pressure generated and the tongue displacement. Different models were tested, each offering varying levels of detail in representing the palate structure. These models range from a simplified approach, with one tissue, to a more intricate representation, involving up to five different tissues, offering a more comprehensive palate model compared to existing literature.

**Results:**

The analysis of results involved examining the distribution of stress on the palate surface, as well as the displacement and forces exerted on the dental crowns. By comparing the obtained results, it was possible to evaluate the precision of the approaches previously described in the literature. The findings revealed that the predictions were less accurate when using the simplified model with a single tissue for the palate, which is the most common approach proposed in the literature. In contrast, the results demonstrated that the palate model with the most intricate structure, incorporating five different tissues, yielded distinct outcomes compared to all other combinations.

**Conclusions:**

The computational methodology proposed, employing the most detailed palate model, has demonstrated its effectiveness and necessity in obtaining accurate data on the impact of non-nutritive sucking habits, which are recognized as a primary contributor to the development of dental malocclusions. In the future, this approach could be extended to conduct similar studies encompassing diverse pacifier designs, sizes, and age groups. This would foster the design of innovative pacifiers that mitigate the adverse effects of non-nutritive sucking on orofacial structures.

**Supplementary Information:**

The online version contains supplementary material available at 10.1186/s12903-023-03848-5.

## Background

Pacifier usage is the most prevalent non-nutritive sucking habit among children. According to a recent systematic review, the prevalence of pacifier use in the assessed countries can reach up to 42.5% by the age of 12 months [4]. Observational and epidemiological studies have established a link between prolonged pacifier use and the development of various types of malocclusions, including open bite, posterior crossbite, and increased overjet, which impact the development of orofacial structures [5]. However, early awareness and identification of the effects of pacifiers on orofacial structures can help prevent the onset or severity of malocclusions [4]. Computational modeling has been extensively employed to support the development of several systems, and, in recent years, its application has been increasingly prominent in the clinical area.

These methods can contribute to expanding our knowledge of the effects of pacifiers on orofacial structures and lead to specific designs that minimize undesirable effects, such as malocclusions.

Although scarce, there are computational studies available in the literature regarding the mechanical behavior of pacifiers in the oral cavity. For instance, in 2007, Levrini et al. [6] aimed to demonstrate the behavior of different types of pacifiers subjected to various pressure levels using a virtual oral cavity model. However, the authors did not provide detailed information on the applied loads, constraints, and the methodology and considerations for developing the model were not adequately substantiated [6]. Nonetheless, it was concluded that pacifier models impact the distribution of stress on the palate, with orthodontic pacifiers being more likely to cause changes in the lateral part while leaving the central part relatively unaffected. The researchers also suggested that the contact area and pressure uniformity are associated with a reduced impact on orofacial structures. However, these conclusions could not be fully supported by the results obtained due to the simplified models used in the study.

Another study [7] aimed to describe the mechanical behavior of three different types of silicone pacifiers on the palate and primary incisors using computational simulation. The geometry of the three pacifiers was obtained through 3D scanning, the palate structure was recreated using computed tomography of a dry skull from a three years old child, and the structure of the tongue was reconstructed using software and data available in the literature. The mechanical properties utilized in the computational models, including the maximum strength of the tongue, were collected from experimental tests. The results obtained led to the conclusion that the orthodontic pacifier induced higher maximum stress on the palate, when compared to the conventional counterpart. Furthermore, the orthodontic pacifier stimulated growth towards the frenum and upward, favoring a more atretic palate. On the other hand, the Super Soothie™ pacifier promoted a more favorable stress distribution on the palate and a stimulatory effect on maxillary growth, both forwards and to the sides. Importantly, the Super Soothie™ pacifier did not influence the inclination of the upper incisors, unlike the conventional and orthodontic pacifiers, which generated displacement stresses in the region of the incisors, could potentially result in an anterior open bite. However, it is worth noting that the computational model developed in this study did not consider different palate tissues with varying mechanical behaviors.

In a recent study [8], the authors aimed to evaluate the mechanical behavior of orthodontic and conventional pacifiers compared to a human nipple model. The analysis of this study revealed significant stresses associated with conventional pacifiers in contact with the oral cavity. On the other hand, it was noted that the orthodontic pacifier exhibited a more pronounced effect like a human nipple, with greater impact on the posterior oral cavity and less strain on soft and hard tissues. However, the study had some limitations, primarily concerning the use of a simplified oral cavity model with homogenous palate tissue.

In two more recent studies [9, 10] the computational models employed provided a more realistic representation of the interaction between the pacifier and palate during the sucking cycle. The objective of the first study [9] was to demonstrate that computational simulation could effectively characterize this interaction by calculating strain, stress, contact force, and contact area. The pacifier model was developed using a hyperelastic, five-parameter Mooney-Rivlin material model, while the palate was represented as a linear elastic material. However, the tongue was not considered in the computational model. To incorporate the tongue effect, a time and spatially varying pressure was applied. To model intraoral negative pressure, a second time-varying periodic pressure was applied. The results obtained from this study indicated that the proposed approach could be utilized in comparative studies to gain further insights into the effects of pacifiers on dental and facial development. The most recent study [10] aimed to follow the methodology proposed in the previous work [9] and compare different pacifier designs. The various pacifier designs were identified based on brand and size and positioned on an age-appropriate palate model. In this study, the authors improved the previous computational model [9] by incorporating a second layer in the palate geometry, representing the mucosa as a linear elastic material. The results obtained from this study allowed the authors to conclude that analyzing the deformation and stress in the contact area between the palate and different pacifiers can provide insights into how the sizing and prolonged interaction between the palate and pacifier may impact palatal growth. Despite the consideration of a more realistic palate model, it is important to note that the actual complexity of the palate is still not fully captured.

Based on the literature review provided above, the analysis of computational studies focused on non-nutritive sucking, as presented earlier, reveals that the models employed to study the effects of pacifiers have simplified representations of the tissue composition of orofacial structures (palate and tongue) [11–14]. Additionally, these models overlook the dynamics of the suction cycle and the negative intraoral pressure [15] generated during suction. Furthermore, the accuracy of these models has not been adequately quantified or discussed.

The main objective of this work is to develop and evaluate a computational model capable of predicting the behavior of the oral cavity during the non-nutritive suction cycle by incorporating details that were lacking in previous studies. The proposed model incorporates a realistic, multi-tissue palate model, allowing for the quantification of stress and displacement induced on the dental tissue due to the loads exerted by a specific pacifier. The computational model developed includes a six-month-old palate comprising various combinations of tissues such as mucosa, cortical bone, cancellous bone, alveolar bone, periodontal ligament, developing teeth, as well as an orthodontic pacifier and tongue. The conducted studies aim to quantify the effect of increasingly detailed palate models to determine the appropriate level of detail required to ensure the representativeness of the model.

## Methods

### Computational model

The computational models used in this study involved a six-month-old palate comprising various combinations of palate tissues, including mucosa, cortical bone, cancellous bone, alveolar bone, periodontal ligament, and developing teeth. At this stage of development, some dental crowns are fully formed and begin to emerge through the bone tissue [12, 16]. By utilizing data from the literature, a computational model was developed to closely resemble the biological structures at this developmental stage. The model had the capability to simulate the effects of any pacifier on orofacial structures, including displacement and loads experienced by the teeth due to pacifier sucking. Additionally, the computational model incorporated the consideration of negative intraoral pressure generated within the oral cavity [9, 10, 15, 17, 18] and tongue displacement [19]. For the studies conducted, two suction cycles were taken into consideration.

To undertake the computational studies, the OpenFOAM® open source library [3], specifically the solids4Foam [1, 2, 20] toolbox, was utilized. In the presented studies, a non-linear total Lagrangian formulation was adopted, following an implicit Euler approach. The mesh density level, time step, and calculation residuals were refined until their impact on the quantities of interest (such as von Mises stress distribution on the palate surface, teeth displacement, and magnitude of the force exerted on the teeth) became negligible. The final meshes employed in the simulations consisted of approximately 2.2 million computational cells.

The preparation of the computational model involves four distinct stages: (i) creation of the geometry; (ii) generation of the computational mesh; (iii) segmentation of the palate and assignment of mechanical properties to the different regions; and (iv) definition of the boundary conditions for the computational model. These stages will be elaborated upon in the subsequent subsections to provide a comprehensive understanding of the followed approach.

Aiming to assess the modelling approach accuracy, four different computational models were studied, each representing a palate with a distinct combination and number of tissues. Considering that malocclusion encompasses various types of misalignments in the teeth and jaws [21], different outcomes were examined. These included the distribution of von Mises stress on the palate surface, the evolution of maximum displacement of the dental crowns, and the forces exerted on each dental crown and by the pacifier on the palate. The aforementioned results were obtained using the post-processing software Paraview [22].

### System geometry

**Fig. 1:**
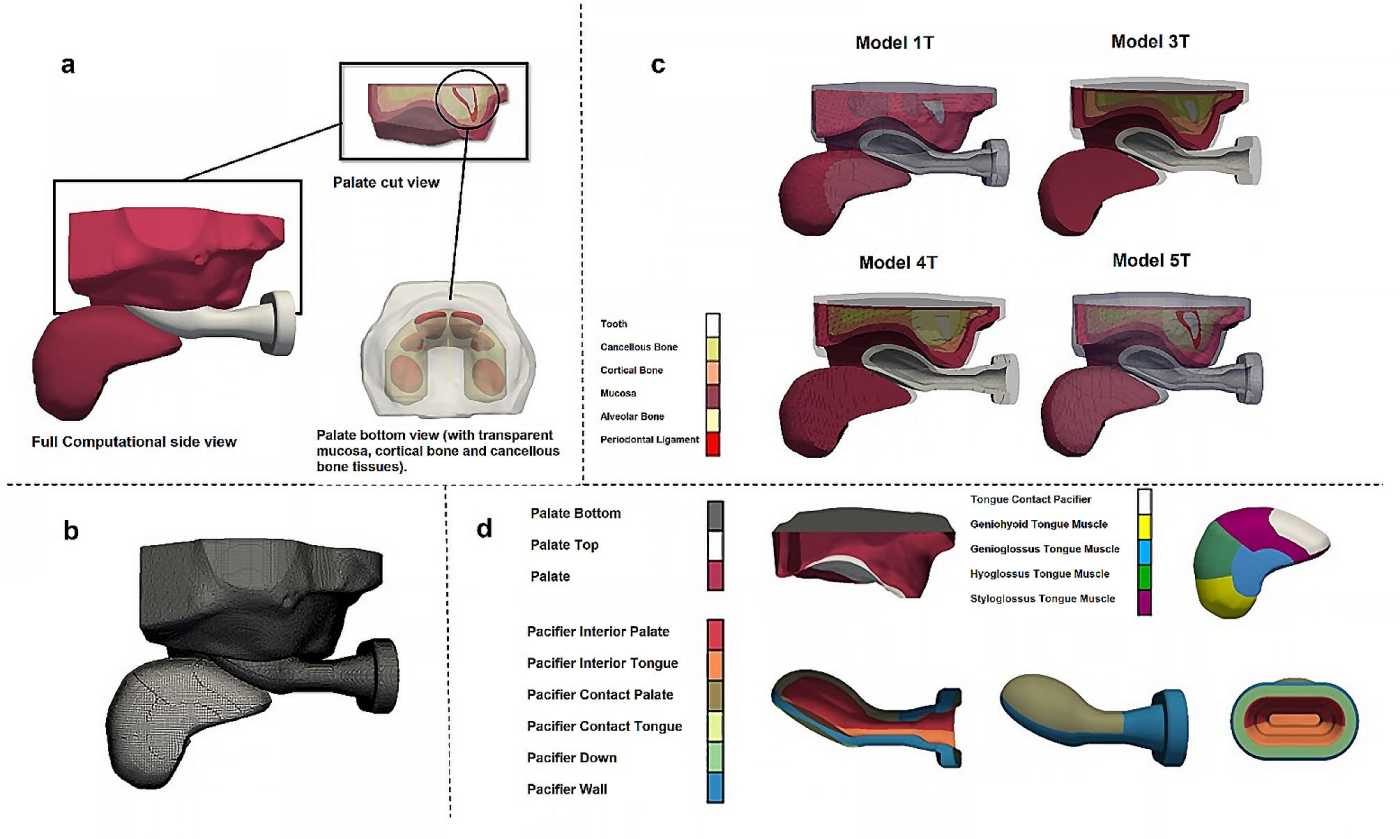
Computational models developed. **a**, Geometry. **b**, Mesh. **c**, Cut view of the four computational models tested, where it is possible to observe the palate tissue regions. **d**, Boundary group faces for each of the three main components - palate, tongue and pacifier

Following the methodology successfully assessed in Atia et al. [23], the external surface of a six-month-old baby’s palate was acquired by scanning a physical model made of plaster with a NobelProcera® 2G System 3D dental lab scanner [24] (see 3D geometry employed is made available on Supplementary Material). Subsequently, the internal details of the palate, which consist of six distinct tissue regions (Fig. 1a) [25], were created using Blender™ software [26], considering the average biological thickness of these tissues, approximately 2 mm for mucosa, cortical bone, and cancellous bone [11, 25].

At the age of six months, the baby’s palate only consists of the crowns of six primary teeth: two central incisors, two lateral incisors, and the first two molars [13, 16, 27]. The data provided in Oka et al. [28] supports the accuracy of the developed models. The geometries of these deciduous teeth crowns were modeled using Blender™ software (Fig. 1a) [26], taking into account their dimensions during the specific developmental phase being studied [29]. Subsequently, the dental crowns were positioned within the palate geometry, considering that at six months, the molars are approximately 28.8 mm apart, and the distance between the right canine and left canine is approximately 41.5 mm [30]. The dental crown geometries were surrounded by a 1 mm thick region representing the periodontal ligament (Fig. 1a) [31]. Furthermore, the teeth and periodontal ligaments were enveloped by the alveolar bone [31]. As there is limited data in the literature regarding the geometry of the alveolar bone at the developmental phase being studied, it was assumed to completely encapsulate the dental crowns and possess an anatomically U-shaped geometry (Fig. 1a) [25].

Figure 1a illustrates the geometry and positioning of the alveolar bone, dental crowns, and their respective periodontal ligaments, as well as the most intricate palate model utilized in this study, highlighting the regions comprising different tissues. The geometry of the NUK Genius Orthodontic Pacifier (Fig. 1) was obtained from the manufacturer, Mapa GmbH, and prepared for simulation using the 3D modeling software Blender™. The pacifier geometry employed in this study corresponds to Size 1, which is appropriate for a six-month-old baby, the specific developmental phase under investigation [32].

The geometry of the tongue was designed to reflect the dimensions specific to the developmental phase being studied, with a length of 3.74 cm and a width of 1.69 cm [33]. The regions corresponding to the associated muscles were delineated based on data obtained from the literature (Fig. 1d) [31]. The 3D model of the tongue was created using Blender™ software [26]. During the suction process, the tongue exhibits characteristic movements facilitated by the contraction of specific muscles. Figure 1 illustrates the developed 3D model of the tongue, highlighting the respective muscle regions.

To establish appropriate boundary conditions (see “Boundary Conditions” subsection below), the outer surface of the model was divided into distinct sub-surfaces: three for the palate, six for the pacifier, and five for the tongue, as depicted in Fig. 1d.

### Computational mesh

The computational mesh (Fig. 1b) was generated using the geometry data exported from Blender™ [26] and provided to the mesh generation software cfMesh [34] from the OpenFOAM® computational library.

In this study, the cartesian mesh workflow available in cfMesh [34] was employed to generate individual computational meshes for the palate, pacifier, and tongue separately. The complete computational mesh, consisting of the three parts, was then constructed by utilizing the OpenFOAM® mergeMeshes utility [3]. Figure 1b presents the computational mesh employed for these studies.

### Palate segmentation and assignment of mechanical properties to different regions

Mechanical properties determine how materials respond to external loads. In the developed computational model, it was necessary to assign appropriate mechanical properties to the different regions/tissues.

For the palate tissues and tongue, the mechanical properties were obtained from the literature, and a linear elastic constitutive model was used, consistent with previous computational studies [6–8].

In this work, the characterization of the pacifier material was performed using a tensile test (ASTM D412), using a rectangular sample cut from a silicone flat sheet provided by the pacifier manufacturer. The engineering stress-strain curve and the linear elastic fit are shown in Additional Fig. 1.

A summary of the mechanical properties for the different palate tissues [11, 12, 35], pacifier, and tongue [36] is provided in Additional Table 1.

The process of developing the computational model involved delineating the different tissues/materials on the computational mesh. An in-house utility, “createCellSetsFromSTL,” was developed to divide the mesh regions based on a geometry file in STL format that delineates the different regions (tissues) where the respective physical properties should be applied. The assignment of these properties in the computational model was performed using the OpenFOAM utility “setMatFromCellZones” [3].

### Boundary conditions

Solids4Foam utility [1, 2, 37] provides various boundary conditions that can be used to impose displacement, traction, or contact conditions.

Based on the system operation and the boundary group faces shown in Fig. 1d, the following boundary conditions were applied:

- “Palate Bottom”: Null displacement condition.
- “Pacifier Down” and “Geniohyoid Tongue Muscle”: Null normal displacement with full slip.
- “Palate”, “Pacifier Wall”, “Hyoglossus Tongue Muscle”, and “Styloglossus Tongue Muscle”: Load-free conditions.
- “Genioglossus Tongue Muscle” (belonging to the tongue): To emulate the suction cycle [15, 17]– [19, 38], cyclic displacement from 0 to 4.2 mm along the vertical direction [19] with a period of 0.39 s [15].
- “Pacifier Interior Palate” and “Pacifier Interior Tongue”: To simulate the oscillatory negative intraoral pressure during the suction cycle, an oscillatory pressure ranging from 0 to 6200 Pa [7, 9, 10, 17, 18] with the same period as the tongue displacement was applied. Additionally, Video 1 demonstrates the synchronization between the peak of tongue displacement and the peak of negative pressure.
- Contact conditions, which adds impenetrability constraints between the two surfaces that make the contact pair [20, 37], were defined to replicate the system operation. One contact condition was considered between the upper boundary of the tongue (“Tongue contact Pacifier”) and the bottom boundary of the pacifier (“Pacifier contact Tongue”). The other contact condition was imposed between the top boundary of the pacifier (“Pacifier contact Palate”) and the bottom boundary of the palate (“Palate Top”). For both contact boundary conditions a friction coefficient of 0.16 [39] was employed.

The tongue model used in the present work is simplified when compared to its complex behavior, which involves several active muscles [31, 40]– [42]. Nevertheless, the inclusion of the tongue with realistic shape and dimensions in this study represents a significant advancement compared to previous literature [6–10]. Furthermore, the simplifications applied to the tongue model do not impact the primary objective of the study, which is to determine the level of detail necessary in the palate model to obtain accurate results.

### Tooth force algorithm

The force vector acting on each tooth tissue was calculated using an integration approach. The force was computed by summing the dot product of the stress tensor and the face normal vector area along the interface surface between the tooth and the neighboring tissues.

## Results

The previous works available in the literature on pacifier assessment have mainly focused on either a single tissue palate [6–9] or only the bone and mucosa [10]. However, the palate of a 6-month-old baby consists of different tissues [13]. Therefore, in this study, four palate models were developed to evaluate and compare the impact of these different tissues on the accuracy of the results. All the models considered in this study include the dental crowns (central incisors, lateral incisors, and first molars) for the specific developmental phase being studied (Fig. 1a and d), which were not included in previous works available in the literature.

The four computational models vary in terms of the number of tissues considered in the palate. Model 1T represents a one-tissue palate, similar to previous studies available in the literature [6–9]. Model 3T includes three palate tissues (mucosa, cortical bone, and cancellous bone), while Model 5T is the most comprehensive, comprising all five known tissues that form the palate (mucosa, cortical bone, cancellous bone, alveolar bone, and periodontal ligament). Model 4T is similar to Model 5T but does not include the periodontal ligament (Fig. 1c). Figure 1c provides a cross-sectional view of the four computational models, illustrating the distribution of the different tissues.

### Palate stress and force exerted on palate

**Fig. 2:**
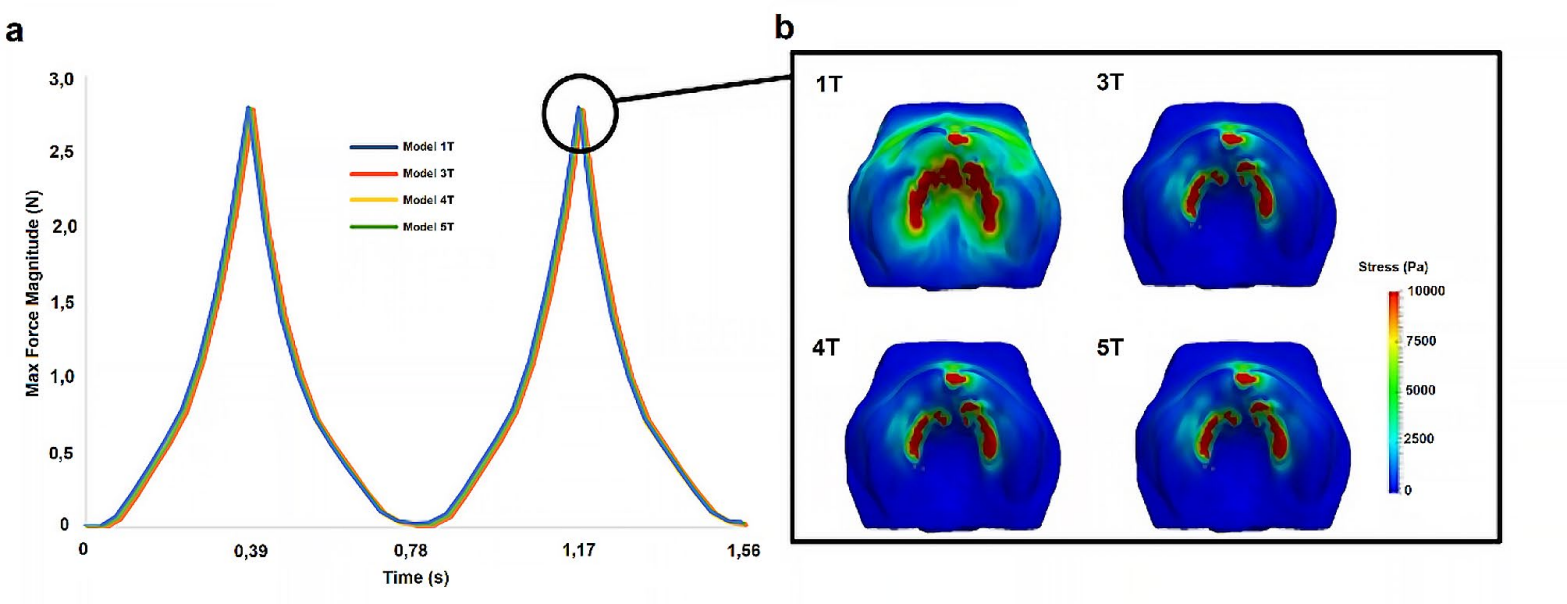
Evolution of the magnitude of the force exerted by the pacifier on the palate over two suction cycles (**e**), all the forces evolutions are almost superimposed, and von Mises stress distribution on the palate (presented in an occlusal projection) for all palate models at the peak of the second cycle (**f**)

The distribution of force and von Mises stresses exerted on the palate for the four palate models studied is shown in Fig. 2a and b, respectively. These results were obtained at the maximum displacement of the tongue during the peak of the second suction cycle (Fig. 2b), representing the highest stresses and maximum force exerted by the pacifier on the palate over the two cycles.

### Tooth displacement

**Fig. 3:**
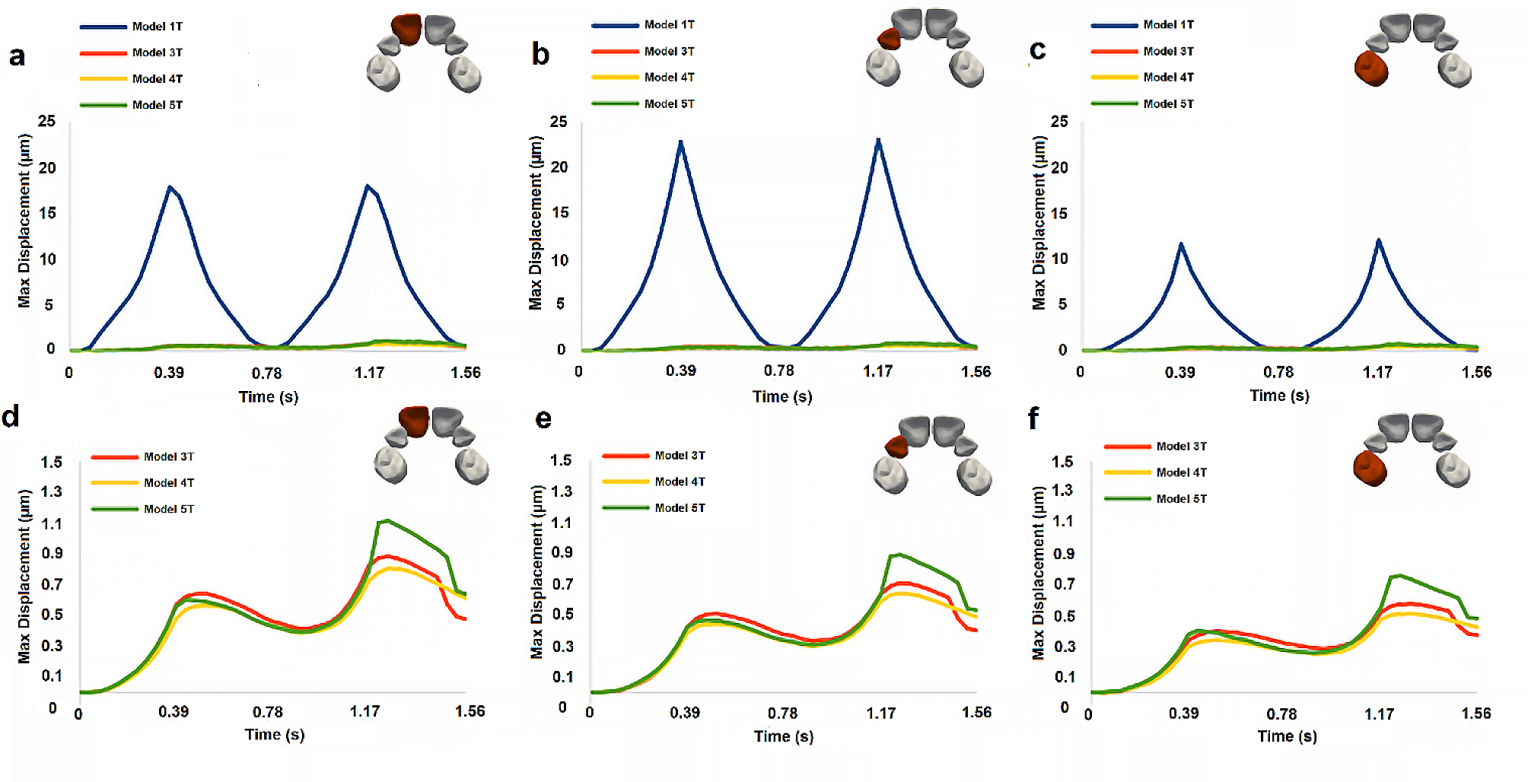
Evolution of maximum displacement magnitude for the right teeth. **a**, **d**, central incisor. **b**, **e**, Lateral incisor. **c**, **f**, First molar. The bottom row presents a zoom for the lower range of the displacement, to allow a better comparison of models 3T, 4T and 5T results

The evolution of the maximum displacement of the teeth on the right side for all palate models (1T-5T) is plotted in Fig. 3. Similar values were obtained for the corresponding left side teeth.

### Tooth force

**Fig. 4:**
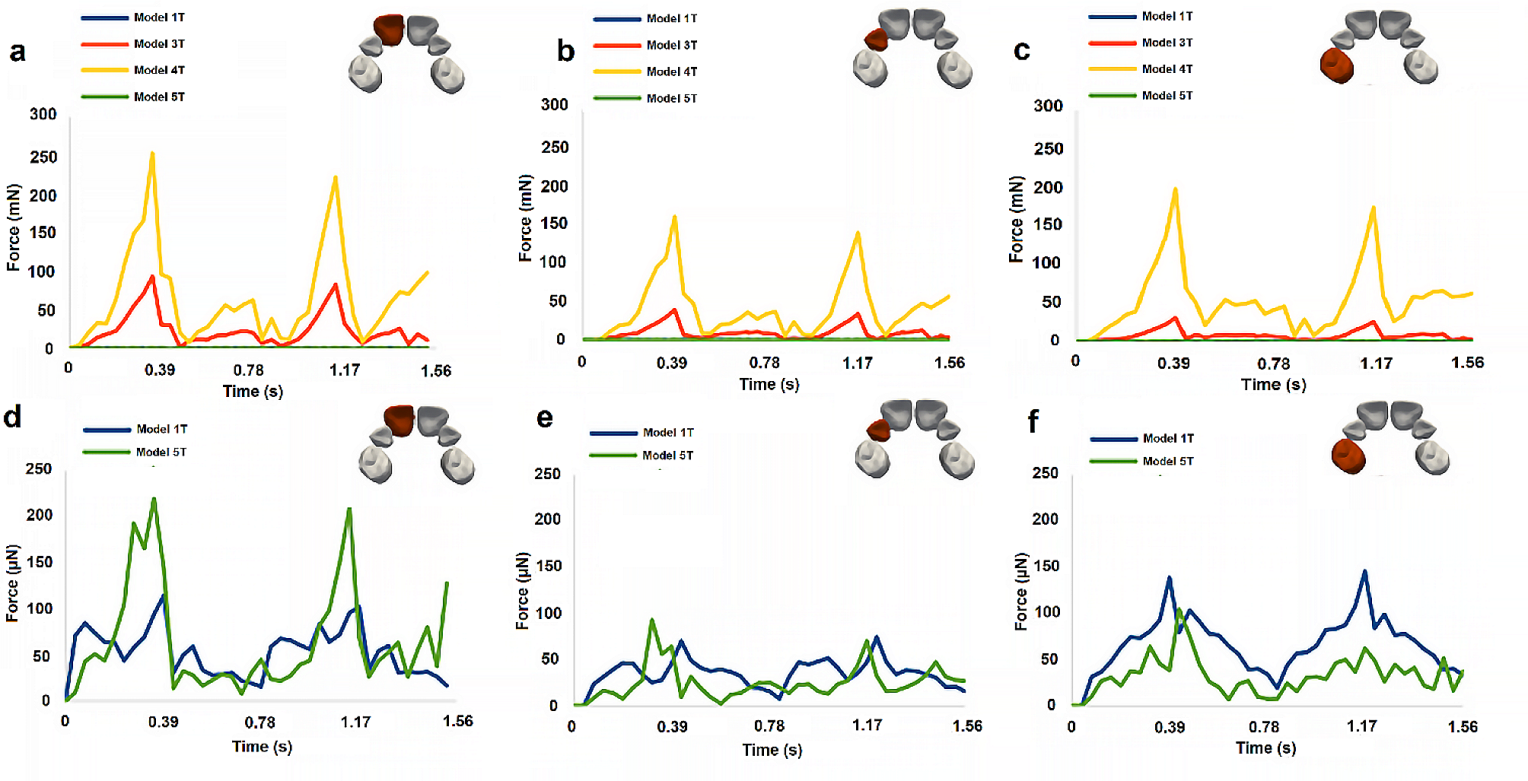
Evolution of the magnitude of the force exerted on the right teeth for the 4 palate models studied. **a**, **d**, central incisor. **b**, **e**, Lateral incisor. **c**, **f**, Fist molar. The bottom row presents a zoom for the lower range of the force magnitude, to allow a better analysis of models 1T and 5T

**Fig. 5:**
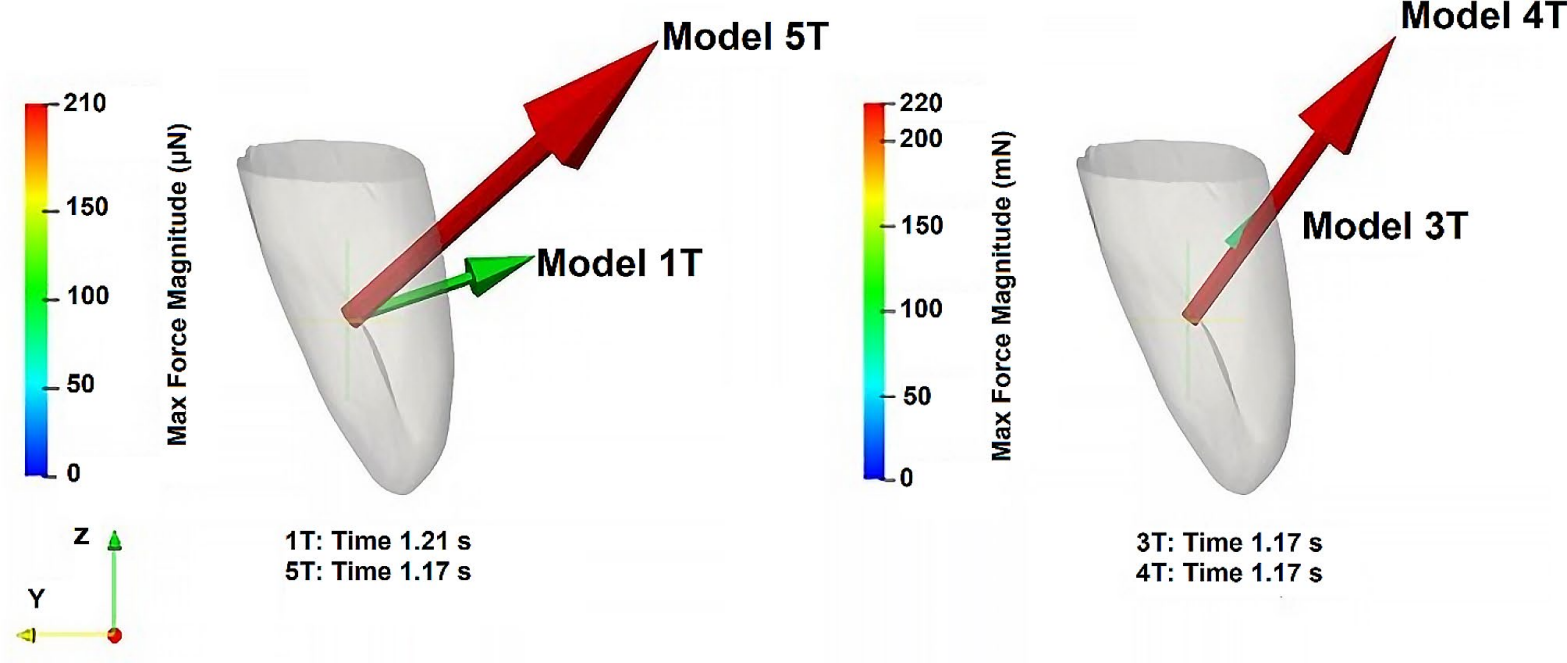
Vectors of the force, corresponding to the instant of the maximum force magnitude, for the palate models 1T and 5T (left) and 3T and 4T (right)

The results of the magnitude of the force exerted on the right-side teeth, computed with the algorithm described in the “Tooth force algorithm” subsection (in the “Methods” section), for all palate models (Models 1T-5T), are plotted in Fig. 4. To complement these results, Fig. 5 illustrates the force vectors corresponding to the maximum force magnitude obtained for all palate models (Models 1T-5T). Similar results were obtained for the corresponding right-side teeth, as mentioned for the displacement.

## Discussion

Based on the results shown in Fig. 2a, all the models considered predict the same evolution of force exerted on the palate. This outcome was expected since the force required to balance the load exerted by the pacifier depends solely on the pacifier and its deformed state, which is similar in all cases. However, the main difference between the different palate models lies in the distribution of stress along the palate surface, as depicted in Fig. 2b. It is evident that this distribution is significantly influenced by the level of detail considered for the palate structure. Specifically, distinct von Mises stress distributions are observed for the palate models, with the highest stress levels obtained for model 1T. Furthermore, the von Mises stress distributions are similar for models 3T, 4T and 5T. These findings emphasize the importance of including the different tissues present in the palate in the simulation, as the simplified models used in previous studies [6–9], equivalent to model 1T, may lead to erroneous conclusions.

Further insights into the effects of the pacifier on orofacial structures can be gained by examining the results of the forces exerted on the teeth (Fig. 4), which in turn influence their displacement (Fig. 3). By individually analyzing the results obtained for each dental crown, it can be concluded that, among the models considered, 5T exhibits larger displacements and magnitudes of force exerted specifically on the central incisors, compared to the other dental crowns. The results obtained for 3T and 4T show similarities to those obtained for 5T in terms of displacement and force magnitude.

In summary, when considering the maximum displacement and magnitude of force exerted on the right central incisor in each model (Figs. 3 and 4), it can be observed that in the 1T model, the effects of the orthodontic pacifier appear to predominantly affect the crowns of the lateral incisors and first molars, while in the 5T model, the incidence is more pronounced on the crowns of the central incisors [see Additional Video 2]. These findings highlight the importance of considering the details of palate morphology, which was not done in previous studies [6–10]. It should be noted that the magnitude of forces and displacements predicted by the model are very small, and to the authors’ knowledge, there are no studies in the literature that directly correlate these force and displacement levels with the development of teeth malocclusions. However, it is widely accepted that prolonged pacifier use is associated with the development of teeth malocclusions. Based on the results obtained in this study, it can be inferred that the cumulative cyclic effect caused by small teeth displacements, occurring approximately 4500 times per hour of pacifier usage, is the most probable cause of teeth malocclusion formation.

Examining the teeth displacement results (Fig. 3) for the different model studies, it can be concluded that the larger displacement observed in 1T, compared to the other models, is contradictory to the similarity in force magnitude predicted by models 1T and 5T (Fig. 4). However, the results presented in Fig. 5 demonstrate that although the force vectors have similar magnitudes in 1T and 5T models, they have different directions. The force predicted by 1T is almost perpendicular to the tooth axis, suggesting a greater deflection of the dental crown due to bending. Conversely, the forces predicted by 3T, 4T, and 5T have a significant component parallel to the dental axis, resulting in a smaller tooth displacement.

Finally, the differences observed between the 4T and 5T models highlight the importance of including the periodontal ligament tissue in the computational model.

## Conclusion

In conclusion, the findings of this study emphasize the importance of considering the detailed morphology of the palate, as well as including the different tissues present in the simulation when studying the effects of orthodontic pacifiers on orofacial structures. The results showed that although all models predicted the same evolution of force exerted on the palate, the distribution of stress along the palate surface and the anterior-occlusal surface varied significantly depending on the level of detail considered for the palate structure. Incorporating different tissues in the palate models resulted in distinct stress distributions, with the highest stress levels observed in the most detailed model.

Furthermore, the study revealed that the forces exerted on the teeth and their resulting displacements varied among the different dental crowns analyzed. The most detailed model exhibited larger displacements and magnitudes of force exerted specifically on the central incisors, when compared with the one applied to the other dental crowns. These findings indicate that the effects of the pacifier are not uniform across all teeth and highlight the importance of considering the specific tooth morphology in such studies.

Although the magnitude of forces and displacements predicted by the model were small, it is widely accepted that prolonged pacifier use is associated with the development of teeth malocclusions. The results of this study suggest that the cumulative cyclical effect of the small displacements suffered by tooth germs, which often occur during pacifier usage, is the most likely cause of dental malposition after tooth eruption.

The comparison of the different models tested also revealed that the inclusion of the periodontal ligament tissue in the computational model had a significant impact on the results, further emphasizing its importance in accurately capturing the effects of non-nutritive sucking habits.

Overall, this study highlights the effectiveness and necessity of employing detailed computational models to obtain accurate data on the impact of orthodontic pacifiers on orofacial structures. The findings provide valuable insights into the distribution of forces and displacements on the palate and teeth, shedding light on the potential mechanisms underlying the development of dental malocclusions. In the future, this approach could be extended to encompass diverse pacifier designs, sizes, and age groups, aiding in the development of innovative pacifiers that mitigate the adverse effects of non-nutritive sucking habits on orofacial structures.

### Electronic supplementary material

Below is the link to the electronic supplementary material.

Supplementary Material 1

Supplementary Material 2

Supplementary Material 3

Supplementary Material 4

## Data Availability

The main data supporting the results in this study are available within the paper and its Supplementary Information. The raw and analysed datasets generated during the study are too large to be publicly shared, yet they are available for research purposes from the corresponding authors on reasonable request.

## Abbreviations

NUK: Natürlich Und Kiefergerecht
3D: Three dimensions or three dimensional
2G: Second-generation
OpenFOAM: Open Field Operation And Manipulation
STL: STereoLithography
