# Supplementary material for: Advancing the assessment of pacifier effects with a novel computational method
Source: BMC Oral Health. 2024 Jan 16;24:87. doi: 10.1186/s12903-023-03848-5 (PMC10792829; doi:10.1186/s12903-023-03848-5)
Supplement: Supplementary file 3 — Supplementary Material 3 [file 12903_2023_3848_MOESM3_ESM.docx]

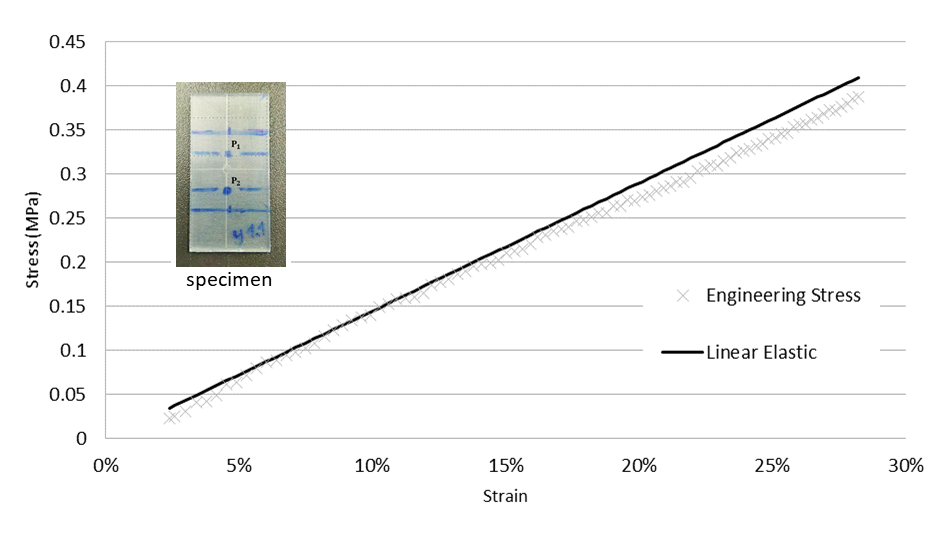


Additional Figure 1. Engineering stress strain curve of the silicone employed in the pacifier: experimental data (measured in a Instron 5969 universal testing machine) and linear elastic fit. The two points marked in the specimen were used to monitor the true deformation with an optical strain gauge.

Additional Table 1. Mechanical Properties of the different regions comprising the computational model

| **Tissues/Materials** | **Mechanical Properties** | | |  |
| --- | --- | --- | --- | --- |
|  | **Density**  **(kg/**$\boldsymbol{m}^{\mathbf{3}}$**)** | **Young’s Modulus**  **(Pa)** | **Poisson**  **Coefficient** | **Reference** |
| Mucosa | 1040 | 1.00$\times{10}^{6}$ | 0.30 | [11] |
| Cortical Bone | 1040 | 1.05$\times{10}^{10}$ | 0.30 | [35] |
| Cancellous Bone | 1040 | 8$\times{10}^{8}$ | 0.30 | [35] |
| Alveolar Bone | 1040 | 1.05$\times{10}^{10}$ | 0.30 | [35] |
| Periodontal Ligament | 1040 | 1.20$\times{10}^{7}$ | 0.45 | [12] |
| Tooth | 1040 | 1.86$\times{10}^{7}$ | 0.31 | [12] |
| Pacifier | 1040 | 1.45 $\times{10}^{6}$ | 0.3 | - |
| Tongue | 1040 | 1.50$\times{10}^{4}$ | 0.49 | [36] |

**Notes:** In preliminary modelling studies it was verified that the maximum equivalent deformation sensed by the pacifier was around 13%, being most of its domain subjected to much smaller values. This justifies the employment of a linear elastic model.

Additional Video 1. Suction cycle calculated and corresponding intraoral pressure evolution.

Additional Video 2. Comparison of the evolution of the displacement calculated for of the right central incisor with Models 1T and 5T (the actual displacement illustrated on the movie has a magnification of 100x).
